# Triterpenoids and Other Non-Polar Compounds in Leaves of Wild and Cultivated *Vaccinium* Species

**DOI:** 10.3390/plants10010094

**Published:** 2021-01-05

**Authors:** Radka Vrancheva, Ivan Ivanov, Ivayla Dincheva, Ilian Badjakov, Atanas Pavlov

**Affiliations:** 1 Department of Analytical Chemistry and Physical Chemistry, University of Food Technologies, 26 Maritza Blvd., 4002 Plovdiv, Bulgaria; radka_vrancheva@yahoo.com; 2 Department of Organic Chemistry and Inorganic Chemistry, University of Food Technologies, 26 Maritza Blvd., 4002 Plovdiv, Bulgaria; ivanov_ivan.1979@yahoo.com; 3 AgroBioInstitute, Agricultural Academy, 8 Dr. Tsankov Blvd., 1164 Sofia, Bulgaria; ivadincheva@yahoo.com (I.D.); ibadjakov@gmail.com (I.B.); 4 Laboratory of Cell Biosystems, Department of Biotechnology, The Stephan Angeloff Institute of Microbiology, Bulgarian Academy of Sciences, 139 Ruski Blvd., 4000 Plovdiv, Bulgaria

**Keywords:** *Vaccinium corymbosum* L., *Vaccinium uliginosum* L., *Vaccinium myrtillus* L., *Vaccinium vitis-idaea* L., GC-MS, HPLC, triterpenes, phytosterols, principal component analysis, hierarchical cluster analysis

## Abstract

The purpose of the current study was to identify and quantify triterpenoids and other non-polar compounds in the leaves of three high bush blueberry cultivars (*Vaccinium corymbosum* L. var. Bluegold, var. Bluecrop and var. Elliott) and three natural populations of *Vaccinium* species (*Vaccinium uliginosum* L., *Vaccinium myrtillus* L. and *Vaccinium vitis-idaea* L.) by means of gas chromatography mass spectrometry (GC-MS) and high-performance liquid chromatography with diode array detector (HPLC-DAD). Metabolite profiles differed significantly among the *Vaccinium* species analyzed, as well as among the populations of the same species. The populations of *V. vitis-idaea* predominantly contained relative concentrations of phytosterols (varying between 10.48% of total ion current (TIC) and 22.29% of TIC) and almost twice the content of triterpenes (from 29.84% of TIC to 49.62% of TIC) of the other berry species investigated. The leaves of *V. corymbosum* varieties biosynthesized the highest relative amount of fatty acids, while the leaves of the populations of *V. uliginosum* had the highest relative concentrations of fatty alcohols. The results of principal component analysis (PCA) and hierarchical cluster analysis (HCA) showed that the diverse populations of each berry species analyzed differed from each other, most likely due to variations in the climatic and geographical conditions of their localities.

## 1. Introduction

In recent years, edible berries of the genus *Vaccinium* (Ericaceae family) have been among the most important berry species for the food and pharmaceutical industries due to their delicious taste and high content of valuable bioactive substances. The genus contains economically important, cultivated and wild berry species, such as blueberries (e.g., *Vaccinium corymbosum* L. (highbush blueberry), *Vaccinium uliginosum* L. (bog blueberry)), bilberry (*Vaccinium myrtillus* L.), and lingonberry (*Vaccinium vitis-idaea* L., also known as cowberry) [1].

*V. myrtillus*, *V. vitis-idaea* and *V. uliginosum* are mostly collected from their natural habitats [2,3,4]. These plant species are of significant economic importance because of the application of their fruits, and sometimes leaves, in the production of various foods, pharmaceuticals, cosmetics, and health-care products. *V. myrtillus* is a perennial, wild and small deciduous shrub that grows in the mountains and forests of Europe [5]. Bilberry has been used since the Middle Ages as an antidiabetic, astringent, antiseptic, and antidiarrheal agent [6]. Crude bilberry fruit extracts are now marketed as pharmaceutical preparations for the treatment of ophthalmological diseases and blood vessel disorders [7]. In European countries, bilberry leaf extracts have traditionally been used as herbal medicines for the urinary tract due to their astringent and antiseptic properties. They also possess antibacterial, anti-inflammatory, hypoglycemic, lipid-lowering and hypolipidemic activities [8,9]. These health benefits are mostly attributed to the various phenolic compounds, such as anthocyanins and phenolic acids [5,10,11]. However, other valuable compounds, such as stilbenes, iridoid glycosides, fatty acids, vitamins, minerals, and dietary fibers have also been found in bilberry leaves [12,13]. The stems and rhizomes of this species are still not well studied organs but they also contain phenolics with various biological activities [3].

*V. vitis-idaea* is a small evergreen shrub. Lingonberry plants are extremely hardy, tolerating 40 °C or lower temperatures, with diverse habitats ranging from lowland to upland and mountain areas, and prefer acid soils [5]. The fruits and leaves of this species also contain a wide range of biologically active compounds: anthocyanins, flavanoids, phenolic acids, chromones, coumarins, lignans, sterols, triterpenoids, fatty acids, minerals (Mg, K, Fe, Cr, Cu and Zn), responsible for their antitussive, anti-inflammatory, anti-catarrhal, and neuroprotective effect in vitro [14,15,16]. Moreover, cowberry fruits and leaves have a well-established role in pharmacognosy and are used in herbal medicine for treatment of urinary- and digestive-tract infections [17]. These studies indicate that the leaves of bilberry and lingonberry are potential raw materials for functional foods.

*V. uliginosum* is a small shrub native to some regions of the Northern Hemisphere, especially at high altitudes, in zones of Europe, Asia and North America. It is an Arctic and boreal circumpolar species, growing on wet acidic soils [5]. While the chemical composition of the fruits of this species (polyphenols, lipids, organic acids, aromatic compounds, mineral elements, vitamins, amino acids and others) have been widely investigated [4,18], studies on its leaf constituents are scanty [5].

*V. corymbosum* var. Bluecrop is a cultivar obtained from four varieties of *V. corymbosum*. The fruits of this plant are widely used especially by the food industry, hence their well-defined chemical constitution (anthocyanins, vitamins (B1, B2, PP, C, A), minerals (Ca, P, Fe) [19].

Although the number of identified phytochemicals in blueberries, bilberry and lingonberry is growing, there is still scanty information regarding their terpenoid content in leaves [2,3,19]. Terpenoids are widely distributed plant secondary metabolites with distinct chemical structures. They produce various aromas and participate in plant defense and pollination [20]. The numerous valuable biological activities (anti-inflammatory, antiulcer, antimicrobial, hepatoprotective, immunomodulatory, hypolipidemic, cholesterol-lowering, antiatherosclerotic, anticarcinogenic, etc.) of these compounds have led to a growing interest in their potential applications in the pharmaceutical, cosmetic and food industries [2]. Thus, there is an increasing demand for naturally occurring triterpenoids, especially for edible and medicinal plants.

Plants are influenced by many abiotic and biotic factors that are a possible reason for the chemodiversity observed among plants of the same species growing in different geoclimates. While the *Vaccinium* fruits have a seasonal nature that implies high harvesting and storage costs, the leaves are available in most seasons and some even in wintertime (e.g., lingonberry and bear berry) [5]. Therefore, the purpose of the current study was to investigate triterpenoids and other non-polar compounds in the leaves of highbush blueberry cultivars (*Vaccinium corymbosum* L. var. Bluegold, var. Bluecrop and var. Elliott), wild bog blueberries (*Vaccinium uliginosum* L.), wild bilberries (*Vaccinium myrtillus* L.), and wild lingonberries (*Vaccinium vitis-idaea* L.) by means of gas chromatography mass spectrometry (GC-MS) and high-performance liquid chromatography with diode array detector (HPLC-DAD), as well as to confirm sample differences or similarities by suitable chemometric analyses, such as principal component analysis (PCA) and hierarchical cluster analysis (HCA).

## 2. Results

### 2.1. Gas Chromatography Mass Spectrometry (GC-MS) Profiling of Non-Polar Compounds in the Leaves of Berry Species

A total of 49 compounds, including hydrocarbons, fatty acids, fatty alcohols, phytosterols and triterpenes, were identified in three wild *Vaccinium* species (*V. uliginosum, V. myrtillus* and *V. vitis-idaea)* and three cultivated varieties of *V. corymbosum* using GC-MS (Table 1).

GC-MS analysis of the *n*-hexane extracts obtained from the leaves of the three *V. corymbosum* cultivars (var. Bluegold, var. Bluecrop and var. Elliot) revealed the presence of 38 phytoconstituents, classified into five categories: hydrocarbons, fatty acids, fatty alcohols, phytosterols, and triterpenes. The species demonstrated high relative content of fatty acids in its leaves (57.5% to 64.4% of total ion current (TIC)), 17 representatives of which were identified (C8 to C26). The most abundant saturated fatty acids (SFA) were palmitic acid C16:0 (9% to 11% of TIC), followed by arachidic acid C20:0 (3% to 4% of TIC). The main polyunsaturated fatty acids (PUFA) were α-linolenic acid C18:3 (29% to 34% of TIC) and linoleic acid C18:2 (9% to 9.7% of TIC) in all analyzed varieties. The PUFA content ranged from 39% to 43% of TIC, but the saturated fatty acids (SFA) were from 18% to 20% of TIC. In the leaf extracts of *V. corymbosum* (var. Bluegold, var. Bluecrop and var. Elliot), margaric acid C17:0 was not identified. The relative amount of fatty alcohols was from 11% to 12.5% of TIC, with nine representatives identified (C8 to C28). The major fatty alcohols were the diterpene alcohol, phytol C20 5.6% to 6.7% of TIC), followed by ceryl alcohol C26 (1.1% to 1.8% of TIC).

Important groups of compounds identified as part of the berry lipids are triterpenes and sterols. Triterpenes were found to compose from 13.0% to 22.6% of the total identified compounds in the leaves of the *V. corymbosum* varieties, while the content of phytosterols varied between 7.3% and 11.0% of TIC. The major phytosterol in the studied berries was β-sitosterol (5.1% to 7.1% of TIC) and the main triterpenes were ursolic acid acetate (4.4% to 12% of TIC), followed by oleanolic acid acetate (3.4% to 6.1% of TIC) and lanosterol (1.2% to 2.2 % of TIC).

GC-MS analysis of the nonpolar extracts obtained from the leaves of three wild populations of *V. uliginosum* (locality Beklemeto, the Balkan Mountains; locality Mount Golyam Perelik, the Rhodope Mountains, and locality Cherni Vrah, Vitosha Mountain) are summarized in Table 1. The populations from the three locations demonstrated high relative content of fatty acids in their leaves (43.1% of TIC to 52.3% of TIC), with 13 representatives identified (from C12 to C26). The main representative of PUFA was α-linolenic acid C18:3 (10.7% to 16% of TIC), while the most abundant SFA was palmitic acid C16:0 (9.4% to 13.8% of TIC), followed by arachidic acid C20:0 (7% to 9% of TIC). The major phytosterol in the populations of *V. uliginosum* was β-sitosterol (7% to 9% of TIC). Ursolic acid acetate (from 6% to 11.8% of TIC) and oleanolic acid acetate (3.3% to 4% of TIC) were the main triterpenes identified. The leaves of population Vu3 from locality Mount Perelik (the Rhodope Mountains) possessed the highest amount of phytosterols and triterpenes compared to the other two populations.

GC-MS based analysis of *n*-hexane leaf extracts of the wild *V. myrtillus* populations revealed the presence of 45 metabolites of different chemical classes (Table 1). Fatty acids were the main nonpolar compounds (between 39.53% of TIC and 53.42% of TIC) in all bilberry samples, the ratio between the PUFA and SFA being in the range of 0.77 (Vm3) and 1.36 (Vm5). The relative concentrations of α-linolenic acid were the highest (between 12.89% of TIC (Vm1) and 22.6% of TIC (Vm5)) of the PUFA identified, the main SFA being palmitic acid (9.46−12.79% of TIC). The relative quantities of triterpenes were between 12.18% of TIC (Vm5) and 27.24% of TIC (Vm1), with domination of β-amyrin, α-amyrin + lupeol, oleanolic acid and ursolic acid. Due to the coelution of α-amyrin and lupeol, their relative amounts were calculated together. The fatty alcohols were in the range of 14.48% of TIC (Vm2) and 23.50% of TIC (Vm3), with phytol being the principal fatty alcohol in all bilberry populations. Phytosterols represented between 9.31% of TIC (Vm2) and 17.78% of TIC (Vm4), with domination of β-sitosterol in all samples of *V. myrtillus.* Hydrocarbons (between 0.42% of TIC (Vm 4) and 0.94% of TIC (Vm2)) and phenolic acids (between 0.41−0.95% of TIC) were found to be in minor relative concentrations compared to the other compounds identified.

The nonpolar extracts of the leaves of the wild-growing populations of *V. vitis-idaea* consisted of 43 metabolites grouped in diverse chemical classes with domination of triterpenes (29.8449.62% of TIC). The major triterpenes in all lingonberry populations were β-amyrin, α-amyrin + lupeol, oleanolic acid, and ursolic acid. The relative quantities of fatty alcohols were in the range of 13.82% of TIC (Vi6) and 19.11% of TIC (Vi4), with phytol being the principal fatty alcohol in all lingonberry samples. Fatty acids represented between 22.50% of TIC (Vi5) and 31.81% of TIC (Vi7) of all non-polar compounds dominated by α-linolenic acid (5.03 to 10.68% of TIC) and palmitic acid (8.21 to 11.36% of TIC). The PUFA:SFA ratio was between 0.52 (Vi5) and 0.92 (Vi1). Phytosterols varied in the range of 10.48% of TIC (Vi5) and 22.29% of TIC (Vi3), with the domination of β-sitosterol (between 4.15% of TIC and 6.88% of TIC), lanosterol (between 2.81% of TIC and 4.96% of TIC), and cycloartenyl acetate (between 0.73% of TIC and 11.69% of TIC). The relative concentrations of hydrocarbons (between 0.21% of TIC and 0.90% of TIC) and phenolic acids (between 0.33% of TIC and 1.35% of TIC) were found to be the lowest compared to the other identified metabolites.

According to the GC-MS data, the metabolite profiles differed significantly among the *Vaccinium* species, as well as among the populations of the same species naturally growing in different environmental conditions (Table 1 and Table 2). Among the species investigated, the leaves of *V. corymbosum* varieties biosynthesized the highest relative amount of fatty acids with prevailing quantity of PUFA, while the leaves of wild-growing populations of *V. uliginosum* had the highest relative concentrations of fatty alcohols and SFA. The leaves of *V. myrtillus* contained the highest relative quantity of hydrocarbons and MUFA, the populations of *V. vitis-idaea* having predominantly relative concentrations of phytosterols and almost twice as much triterpenes as the other berry species. The fatty alcohol 2-hendecanone was identified only in the leaves of the three *V. corymbosum* varieties, 2-tridecanone being present only in the leaves of *V. uliginosum* populations. *n*-Eicosanol was found only in the leaves of *V. corymbosum* and *V. uliginosum* populations. Only the populations of *V. myrtillus* accumulated stigmasterol in their leaves, with the steroid precursor cycloartenol (0.4–0.7% of TIC) found only in the samples of *V. uliginosum.* Capric acid was absent in the leaves of *V. corymbosum* and *V. myrtillus*, and palmitoleic acid and sqalene were not found only in the *V. uliginosum* populations. The leaf extracts of *V. corymbosum* varieties, *V. myrtillus* and *V. vitis-idaea* also contained low relative concentrations of some phenolic acids, most likely released after alkaline hydrolysis was applied.

### 2.2. High-Performance Liquid Chromatography with Diode Array Detector (HPLC-DAD) Quantification of the Main Pentacyclic Triterpenes in the Leaves of Vaccinium Species

In order to obtain more precise data on the quantities of some main pentacyclic triterpenes in the leaves of the *Vaccinium* species, HPLC-DAD analyses were conducted. The estimated content of oleanolic acid, ursolic acid, lupeol and α-amyrin in the leaves of the populations of berry species varied significantly (Table 3). *V. corymbosum* var. Elliott biosynthesized the highest amounts of oleanolic acid, ursolic acid and lupeol (981.3 ± 17.2 µg/g dry weight (DW), 1994.4 ± 25.4 µg/g DW, and 331.1 ± 5.8 µg/g DW, respectively) compared with the other varieties of the same species. *V. corymbosum var.* Bluecrop leaves accumulated nearly twice as much α-amyrin (182.7 ± 3.6 µg/g DW) as the other two varieties. The leaves of *V. uliginosum* (population Vu2) synthesized the highest concentrations of oleanolic and ursolic acid (440.3 ± 7.1 µg/g DW and 1201.3 ± 19.2 µg/g DW, respectively) compared to the other two populations of the same species. The highest quantity of lupeol (319.6 ± 5.6 µg/g DW) was determined in the leaves of population Vu1. α-amyrin was not detected only in the leaves of *V. uliginosum* populations. The highest amount of oleanolic acid, ursolic acid and lupeol in bilberry populations was found in Vm4 (from Mount Perelik), with α-amyrin dominating in Vm1 (from Hut Ambaritza). The highest concentration of oleanolic acid in lingonberry populations was defined in Vi1 (from Mount Perelik at altitude 1930 m), ursolic acid and lupeol being the dominant triterpenes in Vi5 (from locality Beklemeto). The leaves of population Vi7 (from Mount Perelik at altitude 1970 m) accumulated the highest quantity of α-amyrin.

### 2.3. Principal Component Analysis (PCA) and Hierarchical Cluster Analysis (HCA) of GC-MS Data

**Figure 1:**
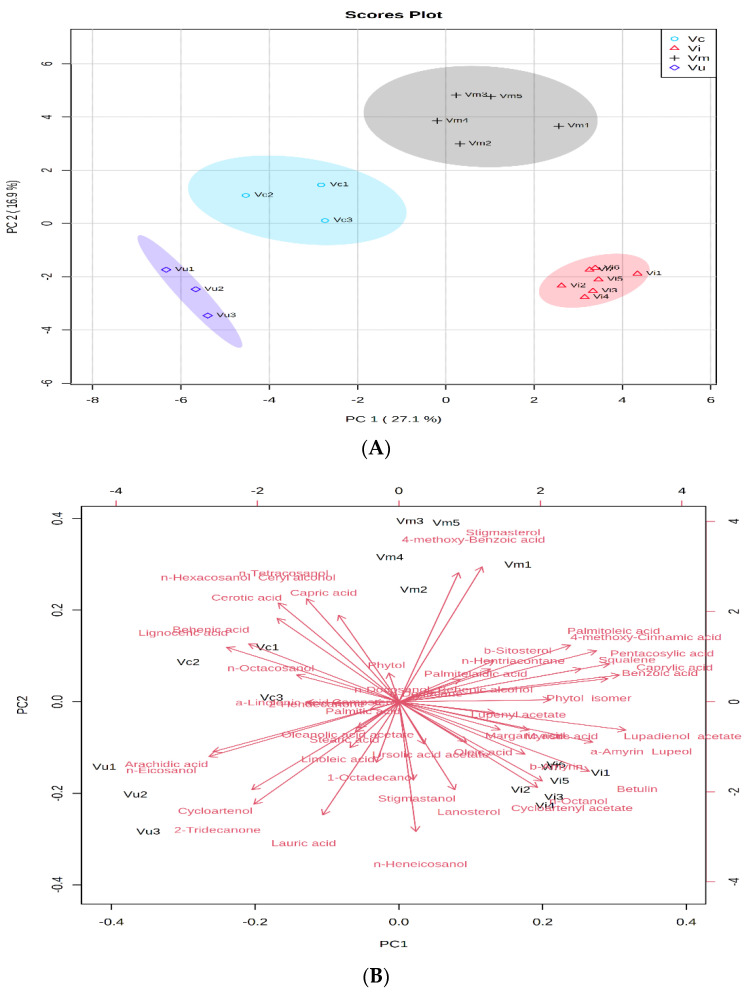
Principal component analysis (PCA) of GC-MS data of metabolites in the leaves of *Vaccinium* species. (**A**) Principal component score plot for the four *Vaccinium* species. (**B**) Eigenvector load values of compounds identified in the four *Vaccinium* species.

In order to establish the differences between the metabolites identified in the berry species, PCA was conducted. According to the resulting PCA plot, the first two principal components PC1 (27.1%) and PC2 (16.9%) accounted for 44% of the total variance of all identified compounds in the leaves of the four *Vaccinium* species (Figure 1A). Metabolites with high positive scores in PC1 were benzoic acid, 4-methoxy-cinnamic acid, caprylic acid, palmitoleic acid, palmitelaidic acid, β- sitosterol, squalene, phytol isomer, ***n***-hentriacontane, and pentacosylic acid that distinguished *V. myrtillus* and *V. vitis-idaea* from the other two species (*V. corymbosum* and *V. uliginosum*), (Figure 1B). Behenic acid, α-linolenic acid, lignoceric acid, ***n***-tetracosanol, ceryl alcohol, octacosyl alcohol, capric acid, and cerotic acid showed high negative load scores in PC1 that distinguished *V. corymbosum* from the other three species (Figure 1B). *V. uliginosum* appeared clearly different from the other berry species by the high negative loadings values of ***n***-eicosanol, arachidic acid, cycloartenol, 2-tridecanone, ***n***-heneicosanol, linoleic acid, and lauric acid in PC2. The positive high scores of ***n***-octanol, cycloartenyl acetate, oleic acid, lanosterol, β-amyrin, α-amyrin and lupeol, lupenyl acetate, betulin and lupadienol acetate clearly distinguished *V. vitis-idaea* from the other three berry species. Stigmasterol and 4-hydroxybenzoic acid had high positive loadings values in PC2 and differentiated *V. myrtillus* from the other species.

The PCA results showed that the different populations of each berry species are relatively similar to each other and each formed a separate cluster, but the type and the relative amount of identified compounds differed among the diverse species investigated.

**Figure 2:**
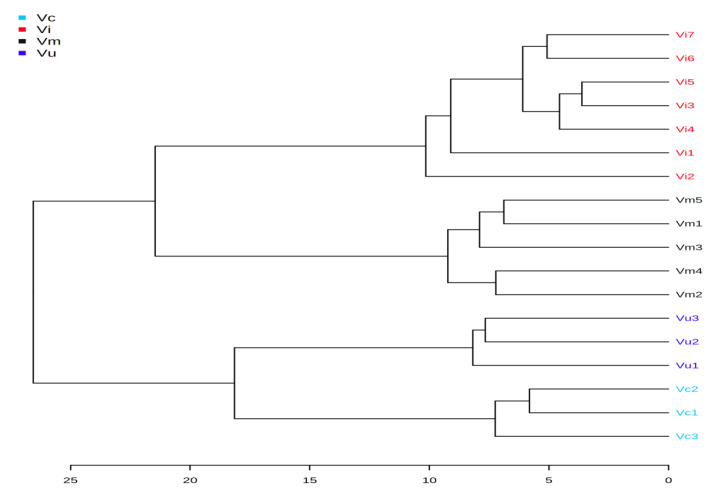
Clustering result of berry species, shown as dendrogram (by Euclidean distance measure, and Ward‘s clustering algorithm).

**Figure 3:**
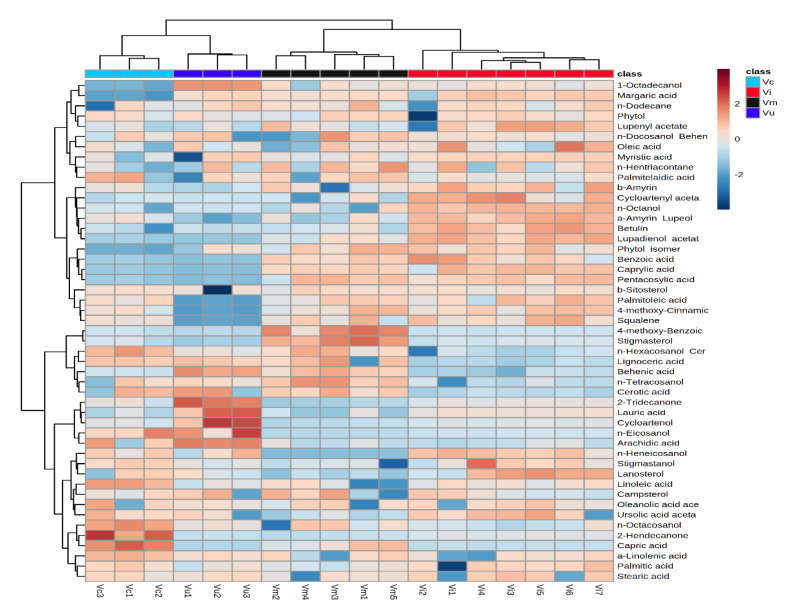
Clustering result of berry species, shown as heatmap. The color scale of the heat map ranged from dark brown (value, + 2) to dark blue (value, –2). The values were normalized by log10 transformation.

HCA was performed to outline the relationships among the eighteen berry samples (Figure 2 and Figure 3). According to the dendrogram and heatmap obtained, the cultivated populations of *V. corymbosum* had the highest phytochemical similarity to the wild-growing populations of *V. uliginosum* and were grouped in one cluster. Wild-growing populations of *V. myrtillus* had the highest similarity to the naturally growing populations of *V. vitis-idaea* and were clustered together. The observed clusters can be explained with the higher relative content of fatty acids and lower relative amount of phythosterols and triterpenes in the leaves of *V. corymbosum* and *V. uliginosum* than in the leaves of *V. myrtillus* and *V. vitis-idaea* populations.

HCA also revealed that the *V. corymbosum* var. Bluegold had higher phytochemical similarity to *V. corymbosum* var. Bluecrop than with *V. corymbosum* var. Elliott and were grouped in one cluster. This differentiation was observed because of the higher relative content of fatty acids and phythosterols and significantly lower relative amount of triterpenes in the varieties Bluegold and Bluecrop than the variety Elliott.

The dendrogram and the heatmap also showed that the blueberry population Vu2 had higher phytochemical similarity to population Vu3 and were grouped in one cluster. Population Vu1 was in a separate cluster due to the lower relative content of fatty acids and higher relative quantity of triterpenes than the other two populations.

The five bilberry populations were grouped in three clusters, with the highest phytochemical distinction observed between populations Vm2 and Vm5. This differentiation was due to the higher relative content of fatty acids and triterpenes, and the significantly lower relative amount of fatty alcohols and phytosterols in the leaves of Vm2 than in the leaves of Vm5. Population Vm2 had the highest phytochemical similarity to Vm4, Vm1 possessing the closest resemblance to population Vm5.

According to the dendrogram and heatmap, the lingonberry populations differed significantly from each other and were grouped in five clusters. The highest phytochemical difference was observed in populations Vi2 andVi7, with Vi3 and Vi5 and Vi6 and Vi7 grouped in two clusters, respectively.

## 3. Discussion

Metabolite profiling of different plant organs provides information on their chemical composition, allows detection of chemically diverse bioactive molecules and unknown compounds, as well as functional information on the metabolic phenotypes of plants [21,22]. The production of particular plant metabolites is a species-specific capability, strongly influenced by climatic and geographical conditions, growth phase, harvest time, post-harvest factors, biotic and abiotic stresses [23,24]. In this context, GC-MS analysis of non-polar leaf compounds of cultivated and naturally growing populations of berry species was conducted. The data obtained and the PCA and HCA performed in the current study showed that metabolite profiles differed significantly among the analyzed *Vaccinium* species, as well as among the populations of the same species naturally growing in different environmental conditions (Table 1, Table 2 and Table 3, Figure 1, Figure 2 and Figure 3). The observed variations are in agreement with other investigations of berry species. Szakiel et al., 2012 [3] reported that the leaves of wild-growing *V. myrtillus* in distinct localities in Finland and Poland possessed significant differences in the triterpenoid content, with lupeol being much more abundant in Polish leaves, while the triterpene ketone, friedelin, was found exclusively in the leaves of Finnish plants, and taraxasterol was detected only in Polish plants. The contents of oleanolic and ursolic acids were similar in Finnish and Polish leaves, but the levels of 2α-hydroxyoleanolic and 2α-hydroxyursolic acids were more than 3-fold higher in the latter [3].

Significant differences have also been established in young and old leaves of wild-growing Finnish and Polish populations of *V. vitis-idaea* [2]. Neutral triterpenes were present in much higher amounts in leaves of Finnish than Polish plants, especially in young leaves collected in August (6-fold higher level). The most abundant compound of this class in Finnish leaves was fernenol (36% and 32% of this fraction in young and old leaves, respectively), which was found in only very small amounts in Polish leaves. The predominant triterpenol in Polish leaves was taraxasterol (32% and 43% of this fraction in young and old leaves, respectively), which in turn occurred in small amounts in Finnish leaves [2].

Linoleic acid (18:2), α-linolenic acid (18:3) and palmitic acid (16:0) were also the major fatty acids in wild-growing lingonberry and bilberry leaves in Poland. [25].

Moreover, the berry species studied here showed a high content of fatty acids (from C8 to C26) in the ***n***-hexane extracts obtained from the leaves. Cultivated blueberries (*V. corymbosum*) biosynthesized the highest concentrations of fatty acids, mainly α-linolenic acid, which is an omega-3 polyunsaturated fatty acid and is essential for humans. The P:S ratio of cultivated blueberries was the highest (2.09 ± 0.11) compared to the wild-growing populations of the species *V. myrtillus* (1.08 ± 0.28), *V. vitis-idea* (0.70 ± 0.16) and *V. uliginosum* (0.52 ± 0.11). This is an important result that shows the potential use of the extracts from the leaves of cultivated blueberries as sources of essential fatty acids. Similar high concentrations of unsaturated fatty acids in the fruits of cultivated blueberries (*V. corymbosum* var. Patriot; var. North blue; var. Duke; var. Chippewa; var. Blue ray; Blue gold and var. Blue crop) were observed in samples of Latvian origin compared to fruits of wild-growing berries. The high content of unsaturated fatty acids can be defined as a chemotaxonomic marker of the species *V. corymbosum* [18].

The main triterpenoids (β-amyrin, α-amyrin, lupeol, oleanolic acid and ursolic acid) and the main phytosterol-β-sitosterol in the leaves of *V. uliginosum*, *V. myrtillus*, *V. vitis-idaea* identified in the current investigation (Table 1 and Table 3) were also in higher concentrations compared with those in plants of Finnish, Polish, Latvian and Chilean populations of the same species [2,3,18,26,27,28].

Triterpenes are part of the plant defense system against different external and internal factors and have an important role in plant metabolism as regulators of the cell processes [2,3,29]. Ursolic, oleanolic acids, lupeol and amyrins are also the main triterpenoids of many fruit species, such as tomato, pepper, orange, grapes [30,31,32,33]. These components are found mainly in the fruit cuticle wax, but similar compounds have also been found in the flowers and leaves waxes of edible honeysuckle (*Lonicera caerulea* var. Kamtschatica) and common heather (*Calluna vulgaris*, Ericaceae) [33,34]. The wax in fruit and leaf cuticles are viewed as being relatively impermeable to gases including water vapor and existing as a cluster of crystalline waxes (mainly ***n***-alkanes, ***n***-alcohols, and fatty acids), both covering and embedded in a matrix of amorphous material (mostly triterpenoids and phytosterols). Water diffusion is considered to occur mostly in the amorphous fraction, while the crystalline cover would prevent further water transport [33,35]. Thus, the established high triterpenes concentration in the analyzed berry population (Table 1 and Table 3) could be explained with the necessity of regulation and protection against various biotic and abiotic stresses. Moreover, ursolic acids and related pentacyclic triterpenoids (oleanolic acids, lupeol, α-amyrin and β-amyrin) have been reported to possess various valuable biological activities, such as antioxidant, anticancer, anti-HIV, antiulcer, anti-inflammatory, antimicrobial, gastroprotective, hepatoprotective, among others [36,37]. The main identified phytosterol-β-sitosterol also revealed diverse bioactivities, such as antidiabetic, chemoprotective, chemopreventive, hypocholesterolemic, anticancer, angiogenic and others [38]. These valuable biological activities are a prerequisite for the potential embedding of berry terpenes and phytosterols in different functional foods, food additives and pharmaceutical products. Furthermore, there is a growing tendency to use agricultural waste resources to produce high-value compounds. *Vaccinium* leaves and stems are considered essentially an agro-waste of the berry industry [39].

## 4. Materials and Methods

### 4.1. Plant material

The leaves of the. *V. corymbosum* (var. Bluegold, var. Bluecrop and var. Elliott), *V. uliginosum*, *V. myrtillus* and *V. vitis-idaea* samples were collected at the same developmental stages before flowering from different localities in Bulgaria, described in Table 4.

### 4.2. Extraction Procedure

The collected leaf samples were lyophilized and ground using an electric mill (Tissue Lyser II, Qiagen) with frequency 30.0 (1/s) at 22 °C for 1 min (three times for 20 s each). Each sample (in triplicate) was extracted twice with acetone (hydro module 1:20, m/v) at room temperature for 24 h, according to the method described by Ivanov et al., 2019 [40]. The combined extracts were evaporated to dryness at 40 °C under vacuum and subjected to alkaline hydrolysis with 2 mol/L KOH (dissolved in 50% ethanol) in water bath at 80 °C under reflux heat for 1.5 h. After cooling, the extracts were neutralized to pH 7 with HCL and separated by liquid-liquid extraction with ***n***-hexane. The ***n***-hexane fractions were evaporated to dryness and used for HPLC-DAD and GC-MS analyses.

### 4.3. GC-MS Analyses and Identification of Compounds

We added 100.0 µL pyridine and 100.0 µL N,O-Bis(trimethylsilyl)trifluoroacetamide (BSTFA) to the dried residue of extracts, then heated on Thermoshaker (Analytik Jena AG, Germany) at 70 °C and 300 rpm for 45 min.

GC-MS analyses were carried out on a 7890A gas chromatograph, interfaced with a mass selective detector 5975C Agilent Technology 5975C inert XL EI/CI MSD (Agilent, USA). Separation of compounds was performed using a DB-5ms silica-fused capillary column (30 m × 0.25 mm × 0.25 μm) coated with 0.25 µm film of polydimethylsiloxane as the stationary phase. The oven temperature program was: initial temperature 100 °C, hold for 2 min, then raise to 180 °C by temperature increments of 15 °C/min, hold at 180 °C for 1 min, then raise by temperature increments of 5 °C/min to 300 °C, and hold for 10 min. The flow rate of the carrier gas (helium) was maintained at 1.0mL/min. The injector and the transfer line temperature were kept at 250 °C. The injection volume was 1 µL in split mode 20:1. All mass spectra were acquired in an electron impact (EI) mode with 70 eV over a mass range of m/z = 50–550.

The mass spectra were read using AMDIS software, version 2.64 (Automated Mass Spectral Deconvolution and Identification System, National Institute of Standardization and Technology (NIST), Gaithersburg, MD, USA). A mixture of aliphatic hydrocarbons C_8_-C_40_ (Sigma) was injected into the system under the above temperature program in order to calculate the retention index (RI) of each compound. The compounds were identified by comparison of their GC-MS spectra and Kovats retention index (RI) with reference compounds in the Golm Metabolome Database [41] and NIST’08 data base (NIST Mass Spectral Database, PC-Version 5.0, 2008 from the National Institute of Standards and Technology, Gaithersburg, MD, USA). The results were presented as % of total ion current (% of TIC).

### 4.4. HPLC-DAD Analysis

The dried extracts were dissolved in methanol (Sigma-Aldrich Chemie GmbH, Darmstadt, Germany). Chromatographic separation and determination of triterpene content was performed on a Hitachi LaChrom Elite^®^ HPLC System (Hitachi High Technologies America, Inc., Schaumburg, Illinois, USA), coupled with diode-array detector (DAD, L-2455) and EZChrom Elite™ software. The separation of oleanolic and ursolic acid (Extrasynthese, Lyon, France) was performed on a reverse-phase column Supelco, Discovery^®^ HS C18 (5 μm, 25 cm × 4.6 mm) operating at 26 °C. Elution was performed with a mobile phase consisted of methanol:0.1% HCOOH = 92:8 (v/v), (Sigma-Aldrich Chemie GmbH, Darmstadt, Germany) in an isocratic mode with a flow rate 0.4 mL/min. The separation of lupeol and α-amyrin (Extrasynthese, Lyon, France) was performed on a reverse-phase column Waters Spherisorb C8 (5 μm, 15 cm × 4.6 mm) operating at 26 °C. Elution was performed with a mobile phase consisted of acetonitrile:0.1% HCOOH = 92:8 (v/v), (Sigma-Aldrich Chemie GmbH, Darmstadt Germany) in an isocratic mode with a flow rate 0.4 mL/min. Detection was carried out at wavelength 210 nm and the sample injection volume was 20 μL for both methods.

### 4.5. Statistical Analysis

PCA and HCA of GC-MS data were conducted using MetaboAnalyst-a web-based platform (www.metaboanalyst.ca) [42]. Firstly, PCA was applied in order to calculate the eigenvector load values and to identify the major statistically different components among the observations (samples). The GC-MS data were mean-centered and the PCA model was obtained at a confidence level of 95%. The GC-MS data were also subjected to HCA that produced a dendrogram by Ward’s method of hierarchical clustering and Euclidean distance measurement between the analyzed samples.

## 5. Conclusions

To the best of our knowledge, this is the first report on metabolite profiling of triterpenes and other non-polar compounds in the leaves of cultivated (*V. corymbosum* var. Bluegold, var. Bluecrop and var. Elliott) and wild populations (*V. uliginosum*, *V. myrtillus* and *V. vitis-idaea*) of *Vaccinium* species growing in Bulgaria. Considering the presence of valuable triterpenes, phytosterols and fatty acids in the leaves of all investigated berry species, they could be used as potential raw materials for production of health foods, food ingredients, as well as active cosmetic ingredients. The established variations in the content of individual triterpenoids and other non-polar compounds among the investigated populations of the same species could be explained by the variations in the climatic and geographical conditions of their habitats. GC-MS based metabolite profiling and subsequent chemometric analyses, such as PCA and HCA, could be successfully applied for the metabolic chemotaxonomy of berry species, which is especially important for the food, pharmaceutical and cosmetic industries. Furthermore, plant material of these species should be analyzed and standardized to ensure that its quality is suitable for specific economic applications.

## Figures and Tables

**Table 1:** Identified phytocompounds in *Vaccinium* spp. analysed by gas chromatography mass spectrometry (GC-MS). The results are given as % of total ion current *.

No.	RI	Compounds	*V. Corymbosum*			*V. Uliginosum*			*V. Myrtillus*					*V. Vitis-idaea*						
			Vc1	Vc2	Vc3	Vu1	Vu2	Vu3	Vm1	Vm2	Vm3	Vm4	Vm5	Vi1	Vi2	Vi3	Vi4	Vi5	Vi6	Vi7
**Hydrocarbons**																				
**1**	1200	*n*-Dodecane	0.12	0.11	0.10	0.14	0.21	0.27	0.32	0.23	0.17	0.19	0.12	0.12	0.20	0.13	0.15	0.07	0.12	0.15
**2**	3100	*n*-Hentriacontane	0.11	0.12	0.21	0.21	0.59	0.25	0.42	0.71	0.66	0.24	0.78	0.41	0.25	0.34	0.12	0.14	0.24	0.32
**Fatty alcohols**																				
**3**	1100	*n*-Octanol	0.56	0.80	0.64	1.15	0.85	1.04	0.80	0.61	0.57	2.54	4.67	4.42	8.94	7.85	7.74	6.69	6.48	7.94
**4**	1308	2-Hendecanone	0.30	0.14	0.19	nd **	nd	nd	nd	nd	nd	nd	nd	nd	nd	nd	nd	nd	nd	nd
**5**	1484	2-Tridecanone	nd	nd	nd	0.88	0.62	0.62	nd	nd	nd	nd	nd	nd	nd	nd	nd	nd	nd	nd
**6**	2152	1-Octadecanol	nd	nd	nd	4.53	4.22	3.85	0.16	0.43	0.41	0.20	0.26	0.11	0.16	0.21	0.24	0.16	0.09	0.16
**7**	2163	Phytol	5.96	5.65	6.71	11.74	7.63	7.84	11.34	8.67	13.29	6.85	11.61	6.26	5.40	7.89	7.55	5.83	4.89	6.72
**8**	2287	Phytol isomer	nd	nd	nd	0.20	0.38	0.33	2.93	1.30	0.44	1.37	2.67	0.23	1.11	0.51	0.93	1.34	0.12	0.25
**9**	2314	*n*-Eicosanol	0.10	0.14	0.10	1.20	1.00	0.74	nd	nd	nd	nd	nd	nd	nd	nd	nd	nd	nd	nd
**10**	2409	*n*-Heneicosanol	1.60	1.25	0.82	0.40	5.20	4.01	nd	nd	nd	nd	nd	2.55	1.52	0.92	1.74	0.67	1.52	1.80
**11**	2545	Behenic alcohol	0.42	0.32	0.15	0.88	0.84	0.60	0.71	0.70	1.88	1.10	0.86	0.33	0.34	0.29	0.41	0.31	0.23	0.29
**12**	2741	*n*-Tetracosanol	0.77	0.57	0.60	1.13	0.81	0.63	1.08	1.61	3.75	3.72	1.52	0.20	0.25	0.12	0.16	0.24	0.15	0.14
**13**	2936	Ceryl alcohol	1.84	1.42	1.09	0.66	0.69	0.53	0.61	1.07	2.34	2.71	0.98	0.24	0.20	0.11	0.18	0.11	0.19	0.22
**14**	3134	Octacosyl alcohol	0.92	1.03	0.72	0.41	0.42	0.38	0.25	0.40	0.82	0.84	0.47	0.17	0.22	0.11	0.16	0.13	0.15	0.26
**Phenolic acids**																				
**15**	1524	4-methoxy-Benzoic acid	nd	nd	nd	nd	nd	nd	0.19	0.15	0.13	0.10	0.12	nd	nd	nd	nd	nd	nd	nd
**16**	1231	Benzoic acid	nd	nd	nd	nd	nd	nd	0.25	0.22	0.09	0.12	0.17	0.68	1.23	0.40	0.14	0.15	0.08	0.10
**17**	1830	4-methoxy-Cinnamic acid	0.12	0.08	0.11	nd	nd	nd	0.51	0.13	0.19	0.18	0.46	0.14	0.12	0.09	0.23	0.23	0.27	0.23
**Fatty acids**																				
**18**	1259	Caprylic acid	nd	nd	nd	nd	nd	nd	0.15	0.11	0.12	0.16	0.11	0.23	0.10	0.18	0.12	0.18	0.12	0.12
**19**	1446	Capric acid	2.03	1.02	0.46	nd	nd	nd	0.14	0.10	0.20	0.10	0.16	nd	nd	nd	nd	nd	nd	nd
**20**	1652	Lauric acid	0.33	0.28	0.19	1.27	3.18	3.75	0.63	0.56	0.26	0.28	0.25	0.48	0.46	0.45	0.52	0.41	0.46	0.47
**21**	1848	Myristic acid	0.70	0.51	0.55	1.00	1.87	2.28	1.68	1.02	0.86	0.64	0.66	1.05	1.05	1.15	1.25	1.07	1.22	1.14
**22**	2017	Palmitoleic acid	0.07	0.25	0.12	nd	nd	nd	0.38	0.11	0.47	0.14	0.31	0.15	0.08	0.36	0.30	0.19	0.47	0.28
**23**	2026	Palmitelaidic acid	0.61	0.60	0.53	0.20	0.47	0.32	0.66	0.62	0.51	0.40	0.63	0.25	0.15	0.17	0.17	0.14	0.13	0.26
**24**	2039	Palmitic acid	9.44	10.94	8.92	9.35	11.44	13.79	11.84	12.79	9.46	10.65	12.18	9.70	10.33	9.35	11.36	8.21	9.48	10.97
**25**	2141	Margaric acid	nd	nd	nd	0.14	0.19	0.22	0.17	0.16	0.15	0.12	0.17	0.14	0.10	0.19	0.26	0.12	0.18	0.16
**26**	2205	Linoleic acid	9.14	8.94	9.68	2.77	4.23	3.92	3.90	7.21	4.01	7.34	5.60	4.04	2.67	3.36	3.06	2.08	3.37	3.74
**27**	2209	Oleic acid	0.22	0.17	0.32	0.74	0.35	0.49	0.46	0.23	0.76	0.25	0.51	0.61	0.36	0.23	0.32	1.60	0.78	0.49
**28**	2218	α-Linolenic acid	34.09	30.45	29.12	10.68	16.03	12.11	12.89	22.25	13.00	16.70	22.62	10.00	6.09	7.74	8.00	5.03	6.84	10.68
**29**	2240	Stearic acid	1.53	1.43	1.57	2.05	2.36	2.69	1.95	1.91	2.54	2.20	1.87	1.60	2.11	2.29	2.07	1.81	2.10	1.93
**30**	2436	Arachidic acid	4.10	2.71	3.61	8.93	6.99	8.21	1.65	2.26	1.13	1.08	1.02	0.56	0.78	0.69	0.74	0.91	1.05	0.64
**31**	2637	Behenic acid	0.27	0.39	0.29	1.42	1.03	1.18	0.83	0.78	1.18	1.04	0.77	0.23	0.27	0.17	0.24	0.27	0.29	0.26
**32**	2833	Lignoceric acid	0.85	1.13	0.93	1.86	1.45	1.31	1.10	1.35	2.96	1.91	1.61	0.22	0.23	0.18	0.26	0.19	0.29	0.23
**33**	2928	Pentacosylic acid	nd	nd	nd	nd	nd	nd	0.27	0.50	0.58	0.56	0.37	0.14	0.09	0.24	0.21	0.11	0.26	0.28
**34**	3032	Cerotic acid	1.05	1.38	1.20	2.72	2.53	2.10	0.82	1.48	2.21	1.18	1.26	0.18	0.18	0.21	0.27	0.18	0.23	0.16
**Phytosterols**																				
**35**	3261	Campesterol	0.21	0.52	0.29	0.76	0.94	1.20	0.22	1.16	1.25	0.71	0.80	0.41	0.54	0.23	0.33	0.19	0.22	0.17
**36**	3279	Stigmasterol	nd	nd	nd	nd	nd	nd	0.31	0.19	0.27	0.15	0.19	nd	nd	nd	nd	nd	nd	nd
**37**	3302	β-Sitosterol	5.98	7.15	5.16	7.99	7.00	9.05	8.94	6.78	12.63	15.51	9.59	6.28	6.88	5.43	4.84	4.15	6.59	5.35
**38**	3314	Stigmastanol	0.55	0.75	0.45	0.58	0.41	0.56	0.47	0.37	0.49	0.51	0.40	0.34	0.24	0.61	2.03	0.45	0.61	0.34
**39**	3318	Cycloartenol	nd	nd	nd	0.70	0.43	0.38	nd	nd	nd	nd	nd	nd	nd	nd	nd	nd	nd	nd
**40**	3403	Lanosterol	1.43	2.19	1.20	2.14	0.77	0.62	0.46	0.23	0.33	0.59	0.21	3.20	4.80	4.32	2.81	4.96	3.44	3.36
**41**	3416	Cycloartenyl acetate	0.26	0.44	0.15	0.42	0.45	0.44	1.74	0.58	0.47	0.30	0.21	4.18	5.24	11.69	8.93	0.73	3.60	4.21
**Triterpenes**																				
**42**	2809	Squalene	0.25	0.43	0.37	nd	nd	nd	1.67	0.56	0.48	1.17	0.22	0.28	0.97	0.29	0.54	1.02	1.29	0.35
**43**	3327	β-Amyrin	1.36	0.43	1.14	0.45	0.38	0.96	11.80	7.55	2.00	2.45	2.38	10.03	3.12	2.61	3.89	6.74	4.40	10.76
**44**	3351	α-Amyrin + Lupeol	3.18	2.16	2.54	0.98	0.29	0.61	6.25	5.41	1.23	0.84	2.11	9.48	11.18	8.79	5.91	12.25	14.81	9.24
**45**	3440	Lupenyl acetate	0.25	0.17	0.21	0.26	0.41	0.25	0.26	1.07	0.45	0.52	0.31	0.16	0.40	0.77	0.34	0.72	0.67	0.44
**46**	3455	Betulin	0.16	0.2	0.28	0.51	0.31	0.42	1.62	0.46	0.79	0.61	0.25	6.58	4.16	1.56	3.91	7.38	9.91	4.87
**47**	3482	Lupadienol acetate	nd	nd	nd	nd	nd	nd	0.72	0.29	0.92	0.14	0.25	3.27	2.91	0.71	1.57	2.91	1.95	3.65
**48**	3502	Oleanolic acid acetate	3.40	4.43	6.15	3.99	3.87	3.28	2.90	2.54	6.33	6.05	3.92	3.20	3.66	3.33	3.05	3.78	2.14	1.85
**49**	3625	Ursolic acid acetate	4.39	7.87	11.96	11.78	6.91	5.90	2.01	1.87	5.27	3.38	2.73	4.73	9.31	11.76	11.38	14.82	6.53	3.50
**Total identified compounds**			98.68	98.58	98.83	97.22	97.75	97.13	98.46	98.70	98.09	97.86	98.34	97.90	98.38	98.06	98.41	98.66	97.96	98.48

* The areas of GC-MS peaks depend not only on the concentration of the corresponding compounds but also on the intensity of their mass spectral fragmentation, so the data given in the table are not true quantification but can be used for comparison of the samples, which was the object of this work; ** nd–not detected.

**Table 2:** Average relative content of chemical classes of compounds identified in *Vaccinium* spp. The results are presented as means ± standard deviation.

Chemical Class	*V. Corymbosum*	*V. Uliginosum*	*V. Myrtillus*	*V. Vitis-Idaea*
Hydrocarbons	0.26 ± 0.05	0.55 ± 0.23	0.77 ± 0.21	0.40 ± 0.12
Fatty alcohols	11.60 ± 0.77	22.15 ± 1.38	19.71 ± 3.65	16.69 ± 2.06
Fatty acids	60.70 ± 3.50	49.21 ± 5.26	45.64 ± 6.04	27.48 ± 3.08
MUFA	0.81 ± 0.05	0.86 ± 0.07	1.00 ± 0.25	0.81 ± 0.46
PUFA	40.48 ± 2.41	16.58 ± 3.43	23.10 ± 1.20	10.96 ± 2.64
SFA	19.41 ± 1.37	31.76 ± 3.45	21.53 ± 0.28	15.71 ± 1.29
PUFA:SFA (P:S) ratio	2.09 ± 0.11	0.52 ± 0.11	1.08 ± 0.28	0.70 ± 0.16
Phenolic acids	0.10 ±0. 02	nd *	0.60 ± 0.24	0.70 ± 0.38
Phytosterols	8.92 ± 1.95	11.61 ± 1.40	13.21 ± 3.38	15.96 ± 3.94
Triterpenes	17.11 ± 4.99	13.85 ± 3.58	18.36 ± 5.70	37.16 ± 6.85

* nd—not detected: MUFA—monounsaturated fatty acids; PUFA—polyunsaturated fatty acids; SFA—saturated fatty acids.

**Table 3:** High-performance liquid chromatography with diode array detector (HPLC-DAD) analysis of the main pentacyclic triterpenes in the leaves of berry species.

Sample, Abbreviation	Oleanolic Acid, µg/g DW	Ursolic Acid, µg/g DW	Lupeol, µg/g DW	α-Amyrin, µg/g DW
*V. corymbosum* var. Bluegold, (Vc1)	735.5 ± 13.6	1102.5 ± 19.3	156.9 ± 2.6	91.7 ± 1.9
*V. corymbosum* var. Bluecrop, (Vc2)	546.4 ± 10.1	1036.3 ± 18.7	73.8 ± 1.2	182.7 ± 3.6
*V. corymbosum* var. Elliott, (Vc3)	981.3 ± 17.2	1994.4 ± 25.4	331.1 ± 5.8	84.6 ± 1.9
*V. uliginosum*, (Vu1)	271.4 ± 4.3	1027.9 ± 18.1	319.6 ± 5.6	0
*V. uliginosum*, (Vu2)	440.3 ± 7.1	1201.3 ± 19.2	236.5 ± 4.1	0
*V. uliginosum*, (Vu3)	310.6 ± 6.4	845.3 ± 15.4	288.2 ± 4.3	0
*V. myrtilus*, (Vm1)	505.2 ± 10.6	424.9 ± 8.5	20.9 ± 0.8	568.2 ± 10.2
*V. myrtilus*, (Vm2)	335.2 ± 6.8	377.9 ± 6.1	20.0 ± 0.8	424.8 ± 8.4
*V. myrtilus*, (Vm3)	643.1 ± 12.2	606.2 ± 12.4	21.1 ± 0.7	102.8 ± 2.1
*V. myrtilus*, (Vm4)	655.8 ± 10.1	815.9 ± 15.5	55.9 ± 1.4	145.0 ± 2.5
*V. myrtilus*, (Vm5)	573.8 ± 9.3	537.0 ± 10.7	35.3 ± 1.1	463.0 ± 12.6
*V. vitis-idaea*, (Vi1)	488.7 ± 8.7	545.5 ± 10.3	432.9 ± 8.2	909.1 ± 17.6
*V. vitis-idaea*, (Vi2)	236.8 ± 4.5	902.5 ± 17.8	300.5 ± 6.4	345.2 ± 7.4
*V. vitis-idaea*, (Vi3)	262.4 ± 4.1	880.5 ± 15.3	604.3 ± 12.1	273.2 ± 6.1
*V. vitis-idaea*, (Vi4)	252.1 ± 4.9	666.7 ± 12.4	769.2 ± 14.6	256.5 ± 5.7
*V. vitis-idaea*, (Vi5)	355.9 ± 6.7	1240.2 ± 21.5	810.2 ± 16.3	494.1 ± 12.4
*V. vitis-idaea*, (Vi6)	202.8 ± 4.8	629.1 ± 12.6	802.5 ± 16.8	377.9 ± 6.8
*V. vitis-idaea*, (Vi7)	357.3 ± 6.6	355.3 ± 5.9	333.2 ± 5.1	1057.9 ± 19.7

**Table 4:** Locations of *V. corymbosum* (var. Bluegold, var. Bluecrop and var. Elliott), *V. uliginosum*, *V. myrtillus* and *V. vitis-idaea* samples.

Sample	ABBREVIATION	Location	Altitude, m	Slope	Date of Collection	Site
*V. corymbosum* var. Bluegold	Vc1	42°18′48.2″ N 23°32′31.1″ E	986	N/NE	4 June 2015	Municipal Forestry Samokov, Rila Mountain
*V. corymbosum* var. Bluecrop	Vc2	42°18′48.2″ N 23°32′31.1″ E	986	N/NE	4 June 2015	Municipal Forestry Samokov, Rila Mountains
*V. corymbosum* var. Bluecrop	Vc3	42°18′48.2″ N 23°32′31.1″ E	986	N/NE	4 June 2015	Municipal Forestry Samokov, Rila Mountain
*V. uliginosum*	Vu1	41°36′30.5″ N 24°35′49.8″ E	1930	N/NW	31 May 2015	Mount Golyam Perelik, Rhodope Mountains
*V. uliginosum*	Vu2	42°34′46.0″ N 23°17′26.2″ E	1950	N/NE	8 June 2015	Aleko Hut, Cherni Vryh path, Vitosha Mountain
*V. uliginosum*	Vu3	42°46′52.4″ N 24°37′21.3″ E	1460	N/NE	30 May 2015	Beklemeto, Balkan Mountains
*V. myrtillus*	Vm1	42°45′18.1″ N 24°46′43.3″ E	1510	N/NE	12 June 2015	Ambaritsa Hut, Balkan Mountains
*V. myrtillus*	Vm2	42°13′53.6″ N 23°35′24.6″ E	1740	N/NE	3 June 2015	Borovets, Musala path, Rila Mountain
*V. myrtillus*	Vm3	41°38′40.6″ N 24°33′23.4″ E	1660	N/NE	1 June 2015	St Marina chapel(near Gela), Rhodope Mountains
*V. myrtillus*	Vm4	41°36′30.5″ N 24°35′49.8″ E	1930	N/NW	31 May 2015	Mount Golyam Perelik, Rhodope Mountains
*V. myrtillus*	Vm5	42°46′27.6″ N 24°37′00.0″ E	1470	N/NE	30 May 2015	Beklemeto, Balkan Mountains
*V. vitis-idaea*	Vi1	41°36′30.5″ N 24°35′49.8″ E	1930	N/NW	31 May 2015	Mount Golyam Perelik, Rhodope Mountains
*V. vitis-idaea*	Vi2	42°34′46.0″ N 24°17′24.2″ E	2060	N/NE	8 June 2015	Aleko Hut, Cherni Vryh path, Vitosha Mountain
*V. vitis-idaea*	Vi3	42°13′53.6″ N 23°35′24.6″ E	1740	N/NE	3 June 2015	Borovets, Musala path, Rila Mountain
*V. vitis-idaea*	Vi4	41°37′58.0″ N 24°33′11.0″ E	1780	N/NE	1 June 2015	near Gradishte Thracian fortress, Rhodope Mountains
*V. vitis-idaea*	Vi5	42°46′27.6″ N 24°37′00.0″ E	1470	N/NE	30 May 2015	Beklemeto, Balkan Mountains
*V. vitis-idaea*	Vi6	42°52′45.2″ N 24°28′58.1″ E	1360	N/NE	11 June 2015	Vasilyov Hut, Balkan Mountains
*V. vitis-idaea*	Vi7	41°36′24.0″ N 24°35′48.3″ E	1970	N/NE	31 May 2015	Mount Golyam Perelik, Rhodope Mountains

## Data Availability

Not applicable.
